# Using proteomics to probe neurons

**DOI:** 10.7554/eLife.09103

**Published:** 2015-07-02

**Authors:** Yunee Kim, Thomas Kislinger

**Affiliations:** Department of Medical Biophysics, University of Toronto, Toronto, Canada; Department of Medical Biophysics, University of Toronto, Toronto, Canada and Princess Margaret Cancer Center, University Health Network, Toronto, Canadathomas.kislinger@utoronto.ca

**Keywords:** alternative splicing, SRM, MRM, synapse, neuroligin, recognition, mouse

## Abstract

Advances in mass spectrometry-based proteomics have allowed researchers to quantify the abundances of the different forms of three closely related proteins in the neurons of mice.

**Related research article** Schreiner D, Simicevic J, Ahrné E, Schmidt A, Scheiffele P. 2015. Quantitative isoform-profiling of highly diversified recognition molecules. *eLife* **4**:e07794. doi: 10.7554/eLife.07794

**Image** The alpha (green) and beta (orange) isoforms of the three neurexin (Nrx 1,2,3) proteins are present at different levels in the mouse brain

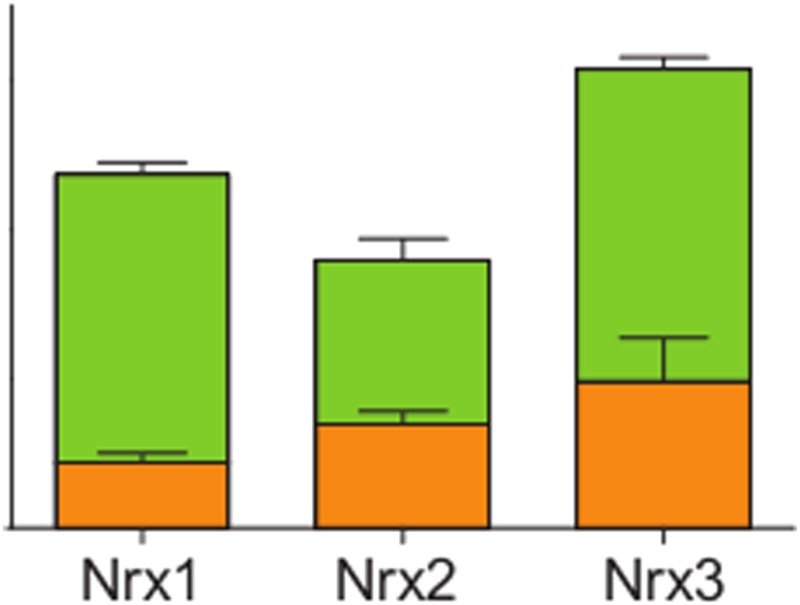

Deconstructing the immensely complex molecular basis of neuronal networks in the mammalian brain would help us to better understand how neurons develop and form connections (called synapses) with each other. Significant strides in the analysis of gene expression have been made in recent years, revealing the regulatory programs that govern the fate of individual neurons, their interactions with diverse ligands, and their ability to adapt to changes, such as environmental cues and injuries (Schreiner et al., 2014). However, measures of gene expression might not reflect the actual levels of proteins produced in cells, so efforts are being made to study the proteins directly.

Evidence suggests that changes in the abundance or activity of proteins in synapses may lead to defects in neurons that are implicated in neuropsychiatric disorders (Craft et al., 2013). Therefore, uncovering the repertoire of proteins produced by neurons could help us understand the underlying molecular basis of such conditions. The large-scale study of the proteins found in cells—known as proteomics—poses significant challenges to researchers. For example, a single gene in a mammalian cell can be used as a template to make many different forms (or ‘isoforms’) of a protein through a process called alternative splicing. These isoforms can be very similar to each other, but they can play very different roles in cells, so it is important for proteomics techniques to be able to distinguish them. The situation is further complicated by the presence of large protein families encoded by very similar genes.

Mass spectrometry is the leading approach for proteomics investigations, and encompasses both global analyses of all the proteins found in cells, and ‘targeted’ approaches that accurately measure the abundance of particular proteins. Selected reaction monitoring (SRM) mass spectrometry is the most widely used technique for targeted proteomics (Elschenbroich and Kislinger, 2011). Proteins are extracted from cells and digested by enzymes to produce millions of fragments (called peptides). However, only a small fraction of these peptides will be unique to the protein or isoform of interest. SRM mass spectrometry is able to accurately determine protein abundance because it can be used to measure just those peptides we are interested in.

The absolute quantification of proteins by SRM involves ‘spiking’ the samples with known concentrations of labelled synthetic peptides after the enzyme treatment (Barr et al., 1996). However, variations in the efficiency of the enzymes can lead to errors with this approach. Alternative approaches use known concentrations of labelled whole synthetic proteins (Brun et al., 2007; Stergachis et al., 2011), which are added to the sample before the enzyme treatment.

However, the use of SRM mass spectrometry to quantify the isoforms of protein families from complex tissues had not been explored. Now, in *eLife*, Peter Scheiffele and colleagues at the University of Basel—including Dietmar Schreiner and Jovan Simicevic as joint first authors—have developed SRM assays to quantify the isoforms of the neurexin family of proteins in the mouse brain (Schreiner, Simicevic et al. 2015).

Neurexins are cell adhesion proteins that play important roles in the formation and differentiation of synapses (Zhang et al., 2010). All three of the genes that encode neurexin proteins contain various segments that can be removed from messenger RNA in different combinations by alternative splicing (Chih et al., 2006). The modified messenger RNA molecules are then translated to make the different neurexin protein isoforms. The segments have been shown to regulate the interactions between ligands and their receptors on the surface of neurons and to alter the activity of synapses in a variety of ways (Aoto et al., 2013).

Schreiner, Simicevic et al. made protein standards that contained different neurexin isoforms fused to the fluorescent protein GFP before carrying out SRM assays (Figure 1). They found that, in several different regions of the brain, neurexin isoforms that contain the segments known as AS3 and AS4 were regulated in the same way, but isoforms that contain another segment called AS6 were regulated independently.

**Figure 1.**
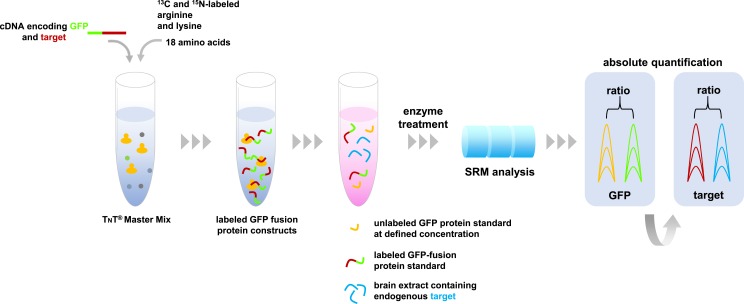
Absolute quantification of proteins by SRM mass spectrometry. A master mix containing the components required for protein synthesis, the amino acids arginine and lysine labeled with carbon-13 and nitrogen-15, and all other unlabeled amino acids are mixed with cDNA molecules that encode the proteins of interest fused with green fluorescent protein (GFP). These fusion proteins (indicated in green-red) are combined with a sample containing the target proteins of interest (depicted in blue), and a known concentration of unlabeled GFP as an internal standard (shown in yellow). This protein mixture is digested by enzymes and the resulting peptides are analyzed by SRM mass spectrometry that specifically measures peptides that are unique to the target proteins. The resulting SRM traces contain information about the abundance of these peptides. The known spiked concentration of the unlabeled GFP standard (yellow) is used to determine the absolute amounts of the labeled GFP fusion protein standard (the green part of the fusion protein). In turn, the target protein component of the GFP fusion protein standard (the red section) is used to determine the absolute amounts of the target proteins in the biological sample (shown in blue).

The absolute quantification of the neurexins revealed that two of the isoforms (called alpha and beta) differed in abundance and that, overall, neurexins are present at relatively high levels across the whole mouse brain. The ability of neurexin isoforms to interact with ligands at synapses was associated with specific segments, which demonstrates that alternative splicing can modulate neurexin activity.

The SRM assays developed by Schreiner, Simicevic et al. enabled them to discriminate between neurexin isoforms that only differed in a single amino acid residue. Their simple approach can be applied to other protein families in any accessible tissue, and hence may be applied to a range of biological questions.
